# Synchronized conductivity modulation to realize broadband lossless magnetic-free non-reciprocity

**DOI:** 10.1038/s41467-017-00798-9

**Published:** 2017-10-06

**Authors:** Tolga Dinc, Mykhailo Tymchenko, Aravind Nagulu, Dimitrios Sounas, Andrea Alu, Harish Krishnaswamy

**Affiliations:** 1 Department of Electrical Engineering, Columbia University, 1300 South West Mudd, 500 West 120th Street, New York, NY 10027 USA; 2 Department of Electrical & Computer Engineering, The University of Texas at Austin, 1 University Station C0803, Austin, TX 78712 USA

## Abstract

Recent research has explored the spatiotemporal modulation of permittivity to break Lorentz reciprocity in a manner compatible with integrated-circuit fabrication. However, permittivity modulation is inherently weak and accompanied by loss due to carrier injection, particularly at higher frequencies, resulting in large insertion loss, size, and/or narrow operation bandwidths. Here, we show that the presence of absorption in an integrated electronic circuit may be counter-intuitively used to our advantage to realize a new generation of magnet-free non-reciprocal components. We exploit the fact that conductivity in semiconductors provides a modulation index several orders of magnitude larger than permittivity. While directly associated with loss in static systems, we show that properly synchronized conductivity modulation enables loss-free, compact and extremely broadband non-reciprocity. We apply these concepts to obtain a wide range of responses, from isolation to gyration and circulation, and verify our findings by realizing a millimeter-wave (25 GHz) circulator fully integrated in complementary metal-oxide-semiconductor technology.

## Abstract (web summary)

Optical non-reciprocity achieved through refractive index modulation can have its challenges and limitations. Here, Dinc et al. introduce the concept of non-reciprocity based on synchronized spatio-temporal modulation of conductivity to achieve different types of non-reciprocal functionality.

## Introduction

Non-reciprocal components are predominantly realized using magneto-optical materials, which are incompatible with integrated-circuit fabrication processes. For this reason, non-reciprocal components today are bulky, expensive and do not find widespread deployment. The ability to integrate magnetic-free non-reciprocal components in modern semiconductor processes would enable exciting frontiers for communications and sensing. Reciprocity can be alternatively broken with active voltage-/current-biased transistors, either in discrete circuits^1^ or embedded within metamaterials^2, 3^. However, the use of active transistors severely limits the linearity and noise performance^4^, and is not an option at optical frequencies. Reciprocity can also be broken using nonlinearities^5–9^. However, nonlinear devices typically exhibit non-reciprocity over a limited range of signal powers, precluding their application in scenarios where linearity to the desired signal is required (e.g., wireless communication). Recently, there has been a strong interest in breaking reciprocity through time-periodic modulation^10–20^, specifically through spatiotemporal modulation of the permittivity of media. In refs. ^10–12^, optical non-reciprocity has been achieved through refractive index modulation with electro-optic phase modulators. However, due to the inherently small amplitude of the refractive index modulation, electro-optic modulators tend to be large, complex, and require high-modulation power levels. In refs. ^13–16^, optical and microwave non-reciprocity have been achieved through spatiotemporal modulation of permittivity in a traveling-wave architecture. At microwave frequencies, permittivity modulation is typically achieved using varactors that exhibit a limited maximum-to-minimum capacitance ratio (*C*_max_/*C*_min_) ranging from 2 to 4, thus leading to large devices. Aside from the aforementioned limitations associated with the small permittivity modulation index, this approach is also associated with unwanted mode/frequency conversion in one direction, necessitating the use of filters or diplexers. Furthermore, permittivity modulation is typically achieved through carrier injection, which is associated with loss, particularly as the frequency of operation increases from the microwave to the millimeter-wave range (>~30 GHz) and beyond. In refs. ^17, 18^, spatiotemporal permittivity modulation was employed in a resonant ring, resulting in angular momentum biasing. The use of localized resonances shrinks the size, but there is a penalty in the bandwidth of operation. Furthermore, in this case as well, permittivity modulation precludes the direct extension of this technique to millimeter-waves.

Here, we introduce the concept of non-reciprocity based on synchronized spatiotemporal modulation of conductivity σ. Conductivity is a variable material property unique to semiconductor media and graphene that enables extremely large modulation index, over a wide range of frequencies, including microwave, millimeter-wave, terahertz and optics. For example, transistors implemented in a complementary metal-oxide-semiconductor (CMOS) process exhibit ON–OFF conductance ratios as high as 10^3^–10^5^ at microwave and millimeter-wave frequencies^21^. Likewise, modulation of graphene conductivity has been shown to enable the realization of optical modulators with unprecedented modulation indices and speeds^22^. Conductivity is commonly associated with loss and absorption through Ohm’s law: the power absorbed at a point with non-zero conductivity, assumed constant in time, is *P*_abs_ = *σ*|**E**|^2^/2, where **E** is the local electric field. However, in the following, we counter-intuitively show that proper control of the temporal variations of conductivity in a circuit can completely suppress absorption and losses, and enable ultra-broadband non-reciprocal functionalities, including gyration, isolation, and circulation. Our approach is inspired by staggered commutated *N*-path switched capacitor filters, which were recently shown to exhibit non-reciprocal phase shift^19^, and is based on adding suitably synchronized conductance modulation sections to transmission lines. Through a rigorous analysis, we show that, by controlling the modulation depth of conductivity sections, we can achieve different types of non-reciprocal functionalities, ranging from non-reciprocal phase shift to ideal isolation. Furthermore, we show that, by including such sections in a ring, we can realize circulators, and provide an experimental demonstration of an integrated CMOS magnet-free circulator for millimeter waves, a first-of-its-kind to the best of our knowledge. Importantly, we show that this approach fundamentally breaks the trade-off between size, operation bandwidth and insertion loss, allowing drastically improved performance at all scales. Beyond opening important directions for wireless communications and radar technology, these principles are also directly extendable to nanophotonic components, and pave the way to the realization of magnet-free photonic topological insulators for strong topological protection and one-way transport.

## Results

### Broadband non-reciprocal phase shifter

**Fig. 1:**
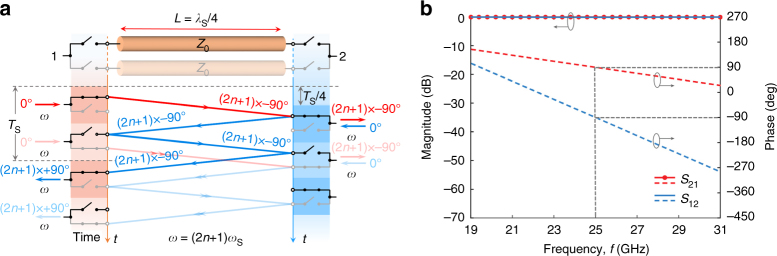
Non-reciprocal phase shifter based on a delay line with switches. **a** Schematic and operation diagram of the non-reciprocal phase shifter, consisting of a transmission line of characteristic impedance *Z*_0_ (which is the same as the port impedance) and delay *T*_s_/4, sandwiched between ideal switches clocked at *ω*_s_ with 50% duty cycle and quarter-period delay between them. This structure provides a non-reciprocal phase shift of −(2*n* + 1)π/2 and −3(2*n* + 1)π/2 for *ω* = (2*n* + 1)*ω*_s_, where *n* = 0, 1, 2, 3, …, corresponding to the case of a gyrator. **b** Scattering parameters for the case with two complementary branches and *f*_s_ = 8.33 GHz. The return losses at both ports are zero for all frequencies. At 25 GHz (*n* = 1), ideal lossless gyrator behavior is observed

Figure 1a sketches the basic principle of operation of the proposed non-reciprocity scheme. It consists of a transmission line segment sandwiched between two time-varying resistors. First, we consider the case in which these resistors are modulated between zero (short circuit) and infinite (open circuit) resistance (i.e., they operate as ideal switches) through periodic square pulses, with the same angular frequency *ω*_s_ (regular frequency *f*_s_) and a 50% duty cycle. The modulation signal of the right switch is delayed with respect to the left one by a value equal to the propagation delay of the transmission line *τ* = *T*_s_/4 (i.e., one quarter of the modulation period *T*_s_). Adding this delay between the two switches allows incident signals from different ports to follow different paths, resulting in non-reciprocal transmission, as illustrated in Fig. 1a. In particular, an arbitrary incident signal from the left-hand side (shown with *red lines*) is transmitted to the other side after one pass through the transmission line. On the other hand, an incident signal from the right-hand side (*blue lines*) needs three passes before it is able to make it through the component and exit at port 1, since in the first two passes it will hit an open circuit termination and experience total reflection. These reflections imply a different delay for signals coming from opposite sides, which results in non-reciprocal transmission phase. For a single branch, this response can be expressed in the time domain as

1 $$v_2^ - (t) = v_1^ + (t \!-\! \tau ){p_1}(t \!-\! \tau ),$$

2 $$v_1^ - (t) = v_2^ + (t \!-\! 3\tau ){p_2}(t \!-\! 3\tau ),$$

where $v_i^ + (t)$ is the incident signal at the *i*-th port, $v_i^ - (t)$ is the output signal at the *i*-th port, and *p*_*i*_(*t*) is the modulation signal of the *i*-th switch. Specifically, *p*_*i*_(*t*) is a periodic 1/0 square-wave signal, with 1 representing the switch in its short-circuit state, allowing unitary transmission, and 0 representing the switch in its open-circuit state, with unitary reflection. By performing a Fourier transform, we find the following expressions for the S-parameters of a single branch:

3 $${S_{21}}(\omega ) = \frac{1}{2}{e^{ - j\omega \tau}},$$

4 $${S_{12}}(\omega ) = \frac{1}{2}{e^{ - j3\omega \tau}},$$

where the factor 1/2 is related to the fact that the switches are closed only for half of the modulation period. Equations (3, 4) highlight the non-reciprocal phase response of the time modulated system. The loss of half of the signal can be easily overcome by adding a second transmission line branch with complementarily clocked switches, shown in a faded fashion in Fig. 1a, which eliminates the factor of 1/2 in Eqs. (3, 4). Importantly, the addition of the second branch also makes the structure impedance matched to *Z*_0_ at all frequencies, realizing an ideal, ultra-broadband non-reciprocal phase shifter. The analytically computed scattering parameters for the two-branch case are shown in Fig. 1b for *f*_s_ = 8.33 GHz. We stress the fact that the bandwidth over which such ideal lossless non-reciprocal phase behavior can be observed is theoretically infinite, and fundamentally limited only by the practical bandwidth of the delay lines and by non-idealities in the switches and their synchronization. For operating frequencies *ω* = (2*n* + 1)*ω*_s_, where *n* = 0, 1, 2, 3, ..., the phases in the forward and backward directions are −(2*n* + 1)π/2 and −3(2*n* + 1)π/2. In this case, the two-branch network operates as an ideal gyrator—a basic non-reciprocal component providing a non-reciprocal phase difference equal to π^23^, which is known to be a building block which can be used to construct arbitrarily complex non-reciprocal networks. In Fig. 1b, the center frequency of 25 GHz corresponds to *n* = 1, and, indeed, at this frequency the structure operates as an ideal gyrator. Given the simplicity and straightforward scalability of this design, the proposed gyrator appears not only as an extremely robust electrical component for millimeter-waves, but also holds exciting potential for nanophotonic systems.

### Isolator

**Fig. 2:**
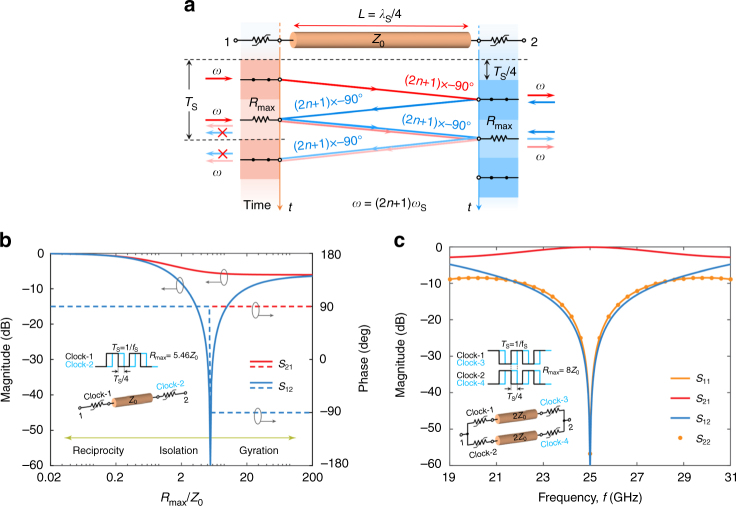
Isolator based on a delay line with time-varying resistors. **a** Schematic and operation diagram of the isolator. The circuit is the same as in the case of the non-reciprocal phase shifter, with the only difference that the time-varying resistors are modulated between 0 and a finite value *R*_max_. **b** Forward (*S*_21_) and reverse (*S*_12_) transmission versus *R*_max_ for *ω* = (2*n* + 1)*ω*_s_. **c** Scattering parameters of a double-branch isolator with complementarily clocked switches (*f*_s_ = 8.33 GHz), exhibiting ideal transmission, matching and isolation. The transmission line impedance is set to *Z* = 2*Z*_0_ and *R*_max_ ≈ 8*Z*_0_. Ideal isolation is achieved at 25 GHz (*n* = 1). The inset shows the corresponding network schematic and clocks

So far we have considered the use of ideally infinite conductivity modulation. Other interesting opportunities arise, however, if the time-varying resistors in Fig. 1 are modulated between zero resistance (short circuit) and a finite resistance ${R_{{\rm{max}}}}$. In this scenario, when the signals hit ${R_{{\rm{max}}}}$, they are partially reflected and partially transmitted (Fig. 2a). This leads to the time-domain evolution

5 $$v_2^ - (t) = v_1^ + (t \!-\! \tau ){p_1}(t \!-\! \tau ) + {T^2}v_1^ + (t \!-\! \tau )[1 \!-\! {p_1}(t \!-\! \tau )],$$

6 $$v_1^ - (t) = Tv_2^ + (t \!-\! \tau ) + {\Gamma ^2}v_2^ + (t \!-\! 3\tau ){p_2}(t \!-\! 3\tau ),$$

and the S-parameters

7 $${S_{21}}(\omega ) = \frac{1}{2}(1 + {T^2}){e^{ - j\omega \tau }},$$

8 $${S_{12}}(\omega ) = T{e^{ - j\omega \tau }} + \frac{1}{2}{\Gamma ^2}{e^{ - j3\omega \tau }},$$

where $T = 2{Z_0}/({R_{{\rm{max}}}} + 2{Z_0})$ is the transmission coefficient between the transmission line and the output ports through the resistor when it is set at ${R_{{\rm{max}}}}$, and ${\rm{\Gamma }} = 1 \!-\! T = {R_{{\rm{max}}}}/({R_{{\rm{max}}}} + 2{Z_0})$ is the corresponding reflection coefficient. Figure 2b shows how the response of the single-branch component at *ω* = (2*n* + 1)*ω*_s_ changes as ${R_{{\rm{max}}}}$ goes from 0 to ∞: for ${R_{{\rm{max}}}} = 0$, there is no modulation and the response is obviously reciprocal. For ${R_{{\rm{max}}}} \to \infty$, we obtain the single-branch gyrator operation described before. For intermediate ${R_{{\rm{max}}}}$, however, |*S*_21_| ≠ |*S*_12_| and the structure shows a range of non-reciprocal responses in phase and amplitude. A particularly interesting response arises for ${R_{{\rm{max}}}} \approx 5.46{Z_0}$, when the contrast between *S*_21_ and *S*_12_ becomes infinite, corresponding to the case of an ideal isolator, i.e., a two-port device that enables unitary transmission in one direction, but full absorption in the opposite one. From a physical point of view, isolation results from the fact that, within one modulation period, half of the signal at *ω* = (2*n* + 1)*ω*_s_ that is transmitted to port 1 after one pass (first term in Eq. (8)) has exactly opposite phase as the other half that is transmitted to port 1 after three passes (second term in Eq. (8)), resulting in destructive interference and isolation at port 1. Most of the power supplied from port 2 is dissipated in the time-varying resistors in their ${R_{{\rm{max}}}}$ state. A small fraction is converted to other frequencies (i.e., intermodulation products), mainly at *ω*_±_ = *ω* ± *ω*_s_, with an amplitude ~−10 dB.

In this configuration, *S*_21_ is relatively low, around −5.4 dB, because for half of the time, the input and output resistors exhibit high resistance. As before, this issue can be easily overcome by adding the second complementary branch. Interestingly, such a two-branch network exhibits near-zero intermodulation products, because they are canceled out by interference between the two paths. However, contrary to the perfectly matched gyrator network, the return losses *S*_11_ and *S*_22_ are not identically 0 but are around −10 dB, as the structure is slightly impedance mismatched due to the finite value of ${R_{{\rm{max}}}}$. It is however possible to overcome this mismatch by changing the impedance of the transmission line sections from *Z*_0_ to *Z* = 2*Z*_0_, and conversely tuning ${R_{{\rm{max}}}}$ to ~8*Z*_0_, allowing simultaneous achievement of ideal isolation, perfect matching and zero insertion loss. This is shown in Fig. 2c for *f*_s_ = 8.33 GHz and 25 GHz operation frequency (*n* = 1). As isolation is the result of destructive interference, it has a finite, yet moderately large, fractional bandwidth of ~36% for *n* = 1, primarily determined by the electrical length of the line. This operation is particularly counter-intuitive, since it shows how the presence of finite losses, through properly synchronized modulation, may be engaged to realize ideal, loss-less, broadband isolation. It is well established that in a two-port device, isolation can be achieved only through the presence of absorption^24^. Through proper synchronization of the spatiotemporal variations of conductivity, absorption takes place only in the reverse path, enabling ideal isolation and zero insertion loss in the forward path.

### Infinitely broadband balanced gyrator

**Fig. 3:**
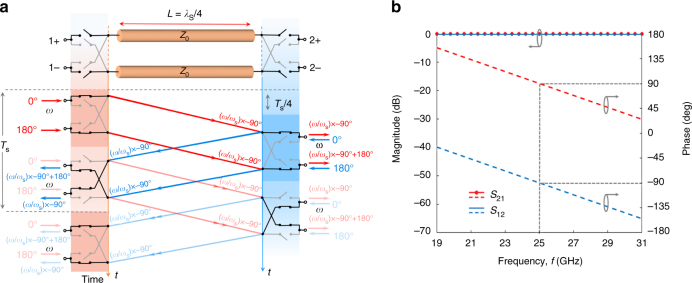
Infinite-bandwidth gyrator based on a balanced pair of delay lines with switches. **a** Schematic and operation diagram of the balanced infinite-bandwidth gyrator, consisting of a pair of transmission lines of characteristic impedance *Z*_0_ (which is the same as the port impedance) and delay *T*_s_/4, sandwiched between ideal switch quads clocked at *ω*_s_ with 50% duty cycle. A pair in the switch quads connects the balanced inputs and outputs to the transmission lines directly in one half cycle, and the crisscrossed pair performs a polarity inversion in the other half cycle. The switch quads on either side operate with quarter-period delay between them. This structure provides a non-reciprocal phase shift of π with zero insertion loss and perfect input/output matching (i.e., ideal gyrator operation) over all frequencies. **b** Scattering parameters for the case of *f*_s_ = 8.33 GHz

The structure of Fig. 1 provides a non-reciprocal phase shift over an infinite bandwidth, yet it operates as an ideal gyrator (with a non-reciprocal phase difference of π) only at the discrete frequencies *ω* = (2*n* + 1)*ω*_s_. An interesting variation of this configuration consists of a balanced pair of switched delay lines, as sketched in Fig. 3a. Here, each port consists of two terminals, differentially fed with a pair of signals equal in magnitude but 180° out of phase. In this case as well, we consider four switches with infinite conductivity modulation on either side, with two switches connecting the inputs/outputs directly to the transmission lines in one half cycle, and the other two (crisscrossed) switches inverting the polarity in the other half cycle. In this configuration, the signals traveling from left to right experience no polarity inversion in the first half cycle, and two polarity inversions that negate each other in the second half cycle. On the other hand, signals traveling from right to left experience a single-polarity inversion in both half cycles. In time domain, this functionality results in

9 $$v_2^ - (t) = v_1^ + (t \!-\! \tau ),$$

10 $$v_1^ - (t) = \!-\! v_2^ + (t \!-\! \tau ),$$

with corresponding S-parameters

11 $${S_{21}}(\omega ) = {e^{ - j\omega \tau }},$$

12 $${S_{12}}(\omega ) = - {e^{ - j\omega \tau }}.$$

Remarkably, these equations, and the corresponding analytically computed S-parameters in Fig. 3b for *f*_s_ = 8.33 GHz, describe an ideal lossless gyrator, with an infinite bandwidth over which the non-reciprocal phase difference is exactly π. This structure confirms that synchronized conductivity modulation in conjunction with balanced operation completely breaks the usual trade-off between size, insertion loss and bandwidth. Ideally, zero insertion loss and infinite bandwidth are possible at arbitrarily small sizes through appropriate increase of the modulation frequency. In practice, loss will be limited by ohmic losses in the transmission lines and switches, the bandwidth by the dispersion effects in the transmission lines and non-idealities in the operation of the switches, and the size by the modulation speed.

### Broadband circulator

**Fig. 4:**
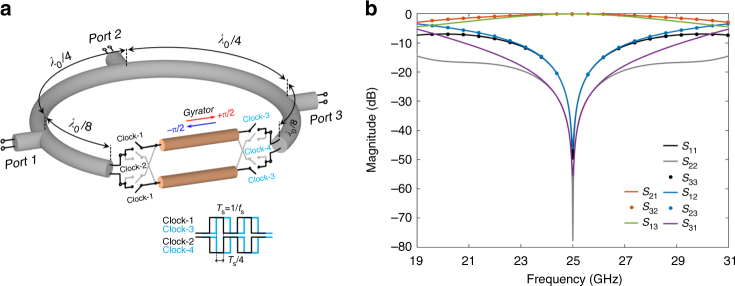
Millimeter-wave circulator based on conductance modulation. **a** Schematic of the circulator: three *λ*/4 transmission line sections with ports in between are wrapped around the ideal gyrator that is shown in Fig. 3a. The gyrator is designed to provide a non-reciprocal ±π/2 phase shift at 25 GHz via modulation at 8.33 GHz. For the sake of symmetry, the gyrator is placed in the middle of one *λ*/4 section. **b** Analytically computed scattering parameters for the circulator

To prove how the proposed scheme for non-reciprocity enables the realization of unique magnetic-free components, we designed and built a millimeter-wave (25 GHz) integrated magnetic-free circulator based on the ideal gyrator network presented in Fig. 3. While ideal gyration is achieved for any modulation frequency as described above, we chose a modulation frequency of 8.33 GHz to miniaturize our device. This frequency approaches the upper limit of the switching speed of the transistor switches available in the 45 nm silicon-on-insulator (SOI) CMOS technology used to build our prototype. As *ω* = 3*ω*_s_, the gyrator exhibits phase shifts of ±π/2, and similar to ref. ^19^, a circulator is realized by wrapping a 3*λ*/4 transmission-line loop around the gyrator component, which is placed symmetrically between port 1 and port 3, see Fig. 4a. The corresponding analytically computed scattering parameters are shown in Fig. 4b (see the Methods section for an analytical technique for computing the scattering parameters of a linear time-periodic network). It is seen that such a circulator, modulated at 8.33 GHz, indeed provides perfect one-way transmission between the ports at 25 GHz in a clockwise direction. The isolation between ports 1, 2, and 3 are not the same, given the asymmetrical topology of the circulator, yet very large isolation is achieved between all ports, a functionality ideal for full-duplex wireless communications and radar.

### Experimental results from a millimeter-wave CMOS circulator

**Fig. 5:**
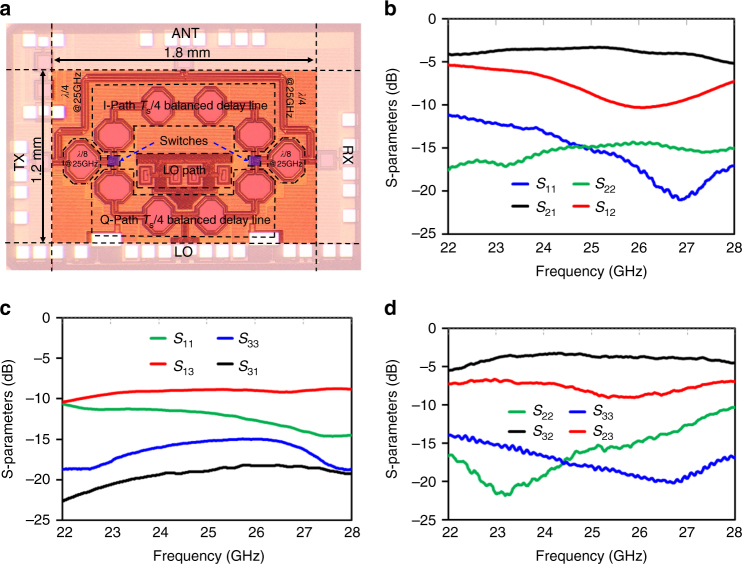
25 GHz 45 nm SOI CMOS circulator S-parameter measurements. **a** Microphotograph of the fabricated circulator. The total size of the 25 GHz circulator is 1.2 mm × 1.8 mm. Measured S-parameters for 8.33 GHz spatiotemporal conductivity modulation between **b** ports 1 and 2, **c** ports 1 and 3, and **d** ports 2 and 3. Low-loss transmission in the clockwise direction (*S*_21_, *S*_32_, and *S*_13_ are −3.3 dB, −3.2 dB, and −8.7 dB, respectively) and large isolation in the reverse direction (*S*_12_, *S*_23_ and *S*_31_ are −10.3 dB, −9 dB, and −18.9 dB, respectively) are measured at the operation frequency of 25 GHz. A broadband isolation of −18.3 to −20.2 dB (limited by the measurement setup) in *S*_31_ is demonstrated over 4.6 GHz bandwidth (the 1 dB insertion loss bandwidth in *S*_21_ and *S*_32_) without any port impedance tuning

**Fig. 6:**
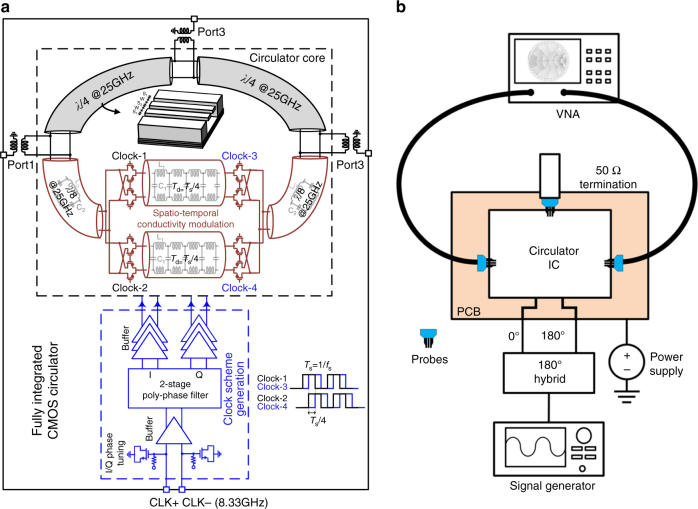
Implementation and measurement of the millimeter-wave CMOS circulator. **a** Block and circuit diagram of the circulator. Non-reciprocal phase response is realized through spatiotemporal conductivity modulation (at 1/3rd of operation frequency) across balanced in-phase and quadrature transmission lines using passive transistor-based in-phase and quadrature switches without direct-current bias. A 3*λ*/4 transmission line loop is wrapped around the non-reciprocal phase element to realize the circulator. The control signals for the spatiotemporal conductivity modulation are generated using on-chip clock generation circuitry. **b** Setup for the measurement of S-parameters. The chip is mounted on a printed circuit board and is probed two ports at a time. The third port is terminated with a third millimeter-wave probe that is terminated with a broadband 50 Ω load

The chip microphotograph and circuit schematic of the 25 GHz 45 nm SOI CMOS circulator are shown in Figs. 5a and 6a, respectively. The design of the circulator is discussed in additional detail in the Methods section. The circulator IC occupies an area of 1.2 mm × 1.8 mm (*λ*/8 × *λ*/6). S-parameter measurements of the 25 GHz circulator were performed using a setup described in the Methods section (Fig. 6b). Under spatiotemporal conductivity modulation at 8.33 GHz configured for circulation in the clockwise direction, broadband strong non-reciprocity is measured (Fig. 5). The measured *S*_21_, *S*_32_, and *S*_13_ transmissions in the clockwise direction are −3.3 dB, −3.2 dB, and −8.7 dB, respectively, at 25 GHz. The measured isolation levels (*S*_12_, *S*_23_, and *S*_31_) in the reverse direction are −10.3 dB, −9 dB and −18.9 dB, respectively, without any port impedance tuning. For wireless communication and radar applications, where the transmitter (port 1) and the receiver (port 3) interface with a common antenna (port 2), the critical transmission parameters are *S*_21_ and *S*_32_, and the most critical isolation is *S*_31,_ which determines the leakage of the transmitter interference to the receiver. Our circulator is designed to exhibit best performance for these three S-parameters. The 1 dB insertion loss bandwidth in *S*_21_ and *S*_32_ is 4.6 GHz (18.4%), and the *S*_31_ isolation over this bandwidth ranges from −18.3 to −20.2 dB. This is notably wideband compared with prior art, including −20 dB *S*_31_ isolation bandwidths of 4.2% in ref. ^19^ and 5.6% in ref. ^18^. The port 1-to-port 2/port 2-to-port 3 input 1 dB compression points are >+21.5/+21 dBm respectively, and are limited by the measurement setup. Port 2-to-port 3 noise figure (NF) is 3.3 to 4.4dB, consistent with the insertion loss and showing negligible degradation due to modulation signal phase noise.

The low-loss levels (~−3 dB) seen in the transmission parameters *S*_21_ and *S*_32_ are the result of parasitic non-zero switch resistance in the ON state and transmission line ohmic loss. It should also be emphasized that the near −20 dB *S*_31_ isolation does not represent the fundamental isolation of the circulator, but rather is limited by reflections at port 2 due to imperfect termination impedance, as is the case with all practical circulators. As can be seen in the measurement setup described in the Methods section, the termination of port 2 is achieved by landing a millimeter-wave probe terminated with a 50 Ω termination impedance. In millimeter-wave measurements, it is challenging to obtain better than -20 dB reflection coefficient from a probe and termination impedance combination. In practice, achieving higher *S*_31_ isolation at millimeter-waves requires the integration of a port 2 impedance tuner on the same chip as the circulator.

## Discussion

When compared with prior work demonstrating non-reciprocal phase shift and a CMOS magnetic-free passive circulator at 750MHz based on staggered commutated *N*-path switched capacitor filters^19^, the ability to perform modulation at a frequency much lower than the operating frequency and the need for only four square-wave clocks with 50% duty cycle, as opposed to eight or more non-overlapping clocks with low duty cycle, significantly simplifies the implementation of the conductivity modulation and the generation of the modulation signals. This is critical in enabling operation at millimeter-waves and in pushing these concepts to higher-frequencies, including photonic circuits. Conversely, *N*-path switched-capacitor circuits do not operate beyond a few GHz in today’s CMOS technologies. Furthermore, the synchronized conductivity modulation across transmission line delays described here results in far more broadband non-reciprocity than ref. ^19^—*N*-path switched-capacitor approaches inherently yield a second-order bandpass filter response with a bandwidth that is directly related to the capacitor value. Such a narrowband filter response is avoided here through the use of transmission lines that are a quarter-wavelength at the modulation frequency.

The concepts and prototype presented in this paper have profound implications for millimeter-wave wireless communication and radar applications. The next generation of cellular communication networks (“5G”) will most likely adopt millimeter-wave technology (specifically, 28 GHz) to meet the increasing demands for data capacity. 28 GHz small-cell base stations will require circulators to transmit to and receive from multiple users simultaneously. Frequency-modulated continuous-wave automotive radars at 77 GHz require circulators to enable simultaneous transmission and reception. These concepts are readily scalable to terahertz and optical frequencies as well, where graphene-based conductivity modulators have recently demonstrated extremely high-modulation indices^22^, opening the door to a new generation of integrated optical non-reciprocal components. Recently, engineering topological order in photonic metamaterials has also drawn significant research interest. Inspired by the exciting discoveries of condensed matter systems exhibiting topological order^25, 26^, active research is underway to demonstrate analogous systems for classical waves (for instance, acoustic^27, 28^ and photonic). To date, however, explorations of photonic topological metamaterials with non-reciprocal responses have relied on magneto-optic effects^29–31^, resulting in the same limitations described earlier. The concepts presented in this paper enable reconfigurable magnetic-free topological photonic metamaterials that potentially revolutionize our ability to control and route electromagnetic waves.

## Methods

### Computing scattering matrix of a linear time-periodic network

A standard way to characterize the response of a two-port time-independent linear network is through its scattering matrix in the frequency domain

13 $$\left( {\begin{array}{*{20}{c}} {v_1^ - } \\ \\ {v_2^ - } \\ \end{array}} \right) = \left[ {\begin{array}{*{20}{c}} {{S_{11}}} & {{S_{12}}} \\ \\ {{S_{21}}} & {{S_{22}}} \\ \end{array}} \right]\left( {\begin{array}{*{20}{c}} {v_1^ + } \\ \\ {v_2^ + } \\ \end{array}} \right),$$

where $v_i^ +$ and $v_i^ -$ are voltage phasors for incoming and outgoing signals from the *i*-th port, respectively, and the scattering matrix is a function of the impinging frequency. If the system is time-modulated with an angular frequency *ω*_s_, there will be mixing between signal and modulation frequencies, and the signals within the system for monochromatic excitation at frequency *ω* can be presented as a superposition of Floquet states:

14 $$v(t) = \mathop {\sum}\limits_{n = - \infty }^\infty {{v_n}{e^{j(\omega + n{\omega _{\rm{s}}})t}}} ,$$

where *v*_*n*_ are the corresponding Fourier amplitudes. In particular, the signals at the input and output ports will be generally represented as in (14).

Using the definition of power waves^32^, and assuming that input and output ports have the same real reference impedance, we can introduce a generalized Floquet scattering parameter ${S_{ij,mn}} = v_{i,m}^ - /v_{j,n}^ +$. Combining the coefficients into vectors, ${\bf{v}}_i^ \pm = \{ v_{i,n}^ \pm \}$, the system (13) can be generalized to a matrix form

15 $$\left( {\begin{array}{*{20}{c}} {{\bf{v}}_1^ - } \\ \\ {{\bf{v}}_2^ - } \\ \end{array}} \right) = \left[ {\begin{array}{*{20}{c}} {{{\bf{S}}_{11}}} & {{{\bf{S}}_{12}}} \\ \\ {{{\bf{S}}_{21}}} & {{{\bf{S}}_{22}}} \\ \end{array}} \right]\left( {\begin{array}{*{20}{c}} {{\bf{v}}_1^ + } \\ \\ {{\bf{v}}_2^ + } \\ \end{array}} \right),$$

where, for example, **S**_11_ is a square Floquet scattering matrix (FSM) consisting of elements *S*_11,*mn*_, which shows the reflection coefficient from the *n*-th to the *m*-th harmonic. These coefficients can be obtained either analytically or numerically from the time-domain response of the system. Representation (15) fully describes the time-varying behavior of the network at all frequencies. It is also consistent with conversion matrix formalism^33, 34^, which is also used for the analysis of LTP systems. From (15), it is evident that such a two-port time-periodic system can be regarded as a network with a number of virtual frequency ports for each physical port. Similar FSMs can be derived for time-modulated systems having  > 2 physical ports.

One can derive a composite FSM of a complex network using, for example, a standard star-product cascading procedure^35^, given that the FSMs of the system’s components are calculated in the same Floquet-state basis. Instead of cascading, here we use another method enabling us to connect multiple FSMs simultaneously. Aggregating FSMs relating all incoming and outgoing waves existing within the system, we can generally write

16 $${\bf{v}}_i^ - = \mathop {\sum}\limits_j {{{\bf{S}}_{ij}}{\bf{v}}_j^ + } .$$

The port indexes in (16) can be split into two subsets corresponding to inner ports further denoted with small letters *m*, *n* and outer ports denoted by capital letters *M*,*N*. Following this, we can split (16) as

17 $$\begin{array}{l}\\ {\bf{v}}_m^ - = \mathop {\sum}\limits_n {{{\bf{S}}_{mn}}{\bf{v}}_n^ + } + \mathop {\sum}\limits_N {{{\bf{S}}_{mN}}{\bf{v}}_N^ + } ,\\ \\ {\bf{v}}_M^ - = \mathop {\sum}\limits_n {{{\bf{S}}_{Mn}}{\bf{v}}_n^ + } + \mathop {\sum}\limits_N {{{\bf{S}}_{MN}}{\bf{v}}_N^ + } .\\ \end{array}$$

The fact that the inner ports are interconnected gives us another equation

18 $${\bf{v}}_m^ - = \mathop {\sum}\limits_n {{{\bf{F}}_{mn}}{\bf{v}}_n^ + } ,$$

where **F** is a highly sparse matrix defining the relations among the modes of the inner ports. For example, if the port 2 is connected to an impedance-matched port 3, we should enforce ${\bf{v}}_2^ - = {\bf{v}}_3^ +$ and ${\bf{v}}_3^ - = {\bf{v}}_2^ +$, leading to **F**_23_ = **F**_32_ = **I**, where **I** is a unitary matrix. Combining the system (17) and Eq. (18), we can find a composite FSM, ${\widetilde {\bf{S}}_{MN}}$, relating only the outer ports, ${\bf{v}}_M^ - = \mathop {\sum}\nolimits_N {{{\widetilde {\bf{S}}}_{MN}}{\bf{v}}_N^ + }$, where

19 $${\widetilde {\bf{S}}_{MN}} = {{\bf{S}}_{MN}} + \mathop {\sum}\limits_n {{{\bf{S}}_{Mn}}\mathop {\sum}\limits_m {\left[ {{{\bf{F}}_{mn}} - {{\bf{S}}_{mn}}} \right]_{nm}^{ - 1}{{\bf{S}}_{mN}}} } ,$$

where [·]^−1^ denotes the inverted matrix. We use Eq. (19) to compute the scattering parameters of the gyrator, isolator, and circulator networks shown in Figs. 1–4.

### Integrated circuit implementation details

The 25 GHz circulator prototype is implemented in the Global Foundries 45 nm SOI CMOS process. A block diagram of the IC is shown in Fig. 6a. The circulator is realized by embedding the balanced non-reciprocal gyrator modulated at 8.33 GHz within a 3*λ*/4 transmission line ring. The balanced non-reciprocal gyrator uses in-phase (0°) and quadrature (90°) branches, resulting in 8 floating-body switches on either side of the in-phase and quadrature balanced delay lines, which are quarter wavelength at the modulation frequency of 8.33 GHz. As mentioned earlier, CMOS technologies offer transistor switches with ON–OFF conductance ratios as high as 10^3^–10^5^, representing practically infinite conductance modulation. The in-phase and quadrature delay lines are miniaturized using four stages of lumped π-type C-L-C sections with a Bragg frequency of ~76 GHz. Ports 1, 2, and 3 are placed along the 3*λ*/4 transmission line in such a manner that the gyrator component is located symmetrically between port 1 and port 3. The *λ*/8 sections on either side of the gyrator are also miniaturized, so that transistor switch capacitive parasitics may be absorbed into the lumped capacitance. The *λ*/4 transmission lines between ports 1 and 2 and ports 2 and 3 are implemented using conductor-backed co-planar waveguides.

The switches are driven with four quadrature clock signals with 50% duty cycle. These clock signals are generated from two input balanced sinusoidal waveforms at 8.33 GHz. A two stage poly-phase filter generates four quadrature signals with 0°/90°/180°/270° phase relationship. The clock generation circuit also allows for phase-imbalance compensation of the in-phase and quadrature clock signals to optimize the circulator performance. After the poly-phase filter, a three stage self-biased CMOS buffer chain with inductive peaking in the final stage generates the square wave clock signals that control the switches.

### S-parameter measurement setup

A diagram of the experimental setup for S-parameter measurements is provided in Fig. 6b. The circulator is tested in a chip-on-printed-circuit-board configuration. All the pads except 25 GHz millimeter-wave ports (namely DC supply, ground, control voltage, and 8.33 GHz clock input pads) are wire bonded to the printed circuit board. An off-the-shelf 180° hybrid is used to generate the balanced (0°/180°) 8.33 GHz clock signals from a signal generator to drive the clock inputs of the implemented circulator. A millimeter-wave probe station and a two-port vector network analyzer are used to measure the S-parameters of the circulator by probing two ports at a time, while a millimeter-wave probe terminated with a broadband 50 Ω termination is landed on the third port. It should be noted that it is hard to obtain better than −20 dB reflection coefficient from a millimeter-wave probe and termination combination.

### Data availability

All relevant data is available upon request.
